# HCC: RNA-Sequencing in Cirrhosis

**DOI:** 10.3390/biom13010141

**Published:** 2023-01-10

**Authors:** Haoyu Wang, Wenjie Shi, Jing Lu, Yuan Liu, Wei Zhou, Zekun Yu, Shengying Qin, Junwei Fan

**Affiliations:** 1 Department of General Surgery, Shanghai General Hospital Affiliated to Shanghai Jiaotong University, School of Medicine, Shanghai Jiao Tong University, Shanghai 200080, China; 2 Bio-X Institutes, Key Laboratory for the Genetics of Developmental and Neuropsychiatric Disorders (Ministry of Education), Shanghai Jiao Tong University, Shanghai 200030, China

**Keywords:** hepatocellular carcinoma, cirrhosis, RNA-sequencing, prognostic signature, fatty acid metabolism

## Abstract

Hepatocellular carcinoma (HCC) ranks the most common types of cancer worldwide. As the fourth leading cause of cancer-related deaths, its prognosis remains poor. Most patients developed HCC on the basis of chronic liver disease. Cirrhosis is an important precancerous lesion for HCC. However, the molecular mechanisms in HCC development are still unclear. To explore the changes at the level of transcriptome in this process, we performed RNA-sequencing on cirrhosis, HCC and paracancerous tissues. Continuously changing mRNA was identified using Mfuzz cluster analysis, then their functions were explored by enrichment analyses. Data of cirrhotic HCC patients were obtained from TCGA, and a fatty acid metabolism (FAM)-related prognostic signature was then established. The performance and immunity relevance of the signature were verified in internal and external datasets. Finally, we validated the expression and function of ADH1C by experiments. As a result, 2012 differently expressed mRNA were identified by RNA-sequencing and bioinformatics analyses. Fatty acid metabolism was identified as a critical pathway by enrichment analyses of the DEGs. A FAM-related prognostic model and nomogram based on it were efficient in predicting the prognosis of cirrhotic HCC patients, as patients with higher risk scores had shorter survival time. Risk scores calculated by the signature were then proved to be associated with a tumor immune environment. ADH1C were downregulated in HCC, while silence of ADH1C could significantly promote proliferation and motility of the HCC cell line.

## 1. Introduction

Liver cancer is the sixth most common cancer and the third leading cause of cancer-related death worldwide causing more than 830,000 deaths in 2020 [1], among which hepatocellular carcinoma (HCC) accounts for about 90% of all primary liver malignancies [2]. In the pathogenesis of HCC, chronic hepatitis B (HBV) is one of the important causes, followed by hepatitis C (HCV) and alcohol abuse. Despite the success of direct-acting antiviral therapy in curing chronic HCV infection, current antiviral therapy can only reduce, rather than eliminate HBV [3].

With advances in gene chips and high-throughput sequencing, several prognostic gene signatures have been established to predict overall survival of HCC [4,5,6]. Liver cirrhosis is usually considered as precancerous lesion of HCC, with 80–90% of HCC cases having underlying cirrhosis [7], and 2–4% patients with cirrhosis will develop HCC annually [3]. The incidence of HCC is a process of a gradual change from normal liver, cirrhotic liver to cancer, however, the detailed mechanism of cirrhosis to HCC is unclear. High-throughput sequencing technologies are now routinely being applied to a wide range of important topics in tumor medicine, often allowing physicians to address tumor evolution that was not possible before.

In the present study, we screened and verified HCC-associated genes combined with self-building RNA sequencing data and public databases. We found fatty acid metabolism (FAM)-related DEGs enriched in HCC development and constructed a FAM-related prognostic signature of HCC. Fatty acids (FA), the main components of several lipid species, are required for energy storage, membrane proliferation, and the generation of signaling molecules [8]. Cancer cells prefer de nove FA biosynthesis rather than exogenous sources, which is more common in normal cells, to meet the demand of their rapid growth [9]. The liver is the central organ of lipid metabolism. Several papers have clarified the role of FA metabolism and related genes in HCC progression [10,11]. Abnormal FA metabolism were also reported to take part in chronic liver diseases [12,13]. In addition, we choose ADH1C, one of the FAM-related prognostic signatures, as a typical example to validate its function in HCC development with in vitro experiments, as well as to explore the potential mechanisms underlying abnormal FA metabolism in the process from cirrhosis to HCC.

## 2. Materials and Methods

### 2.1. Patients and Samples

A total of 18 samples from 13 patients were collected for RNA sequencing, including 8 cirrhosis tissues, 5 pairs of HCC tissues and adjacent paracancerous tissues. Paracancerous tissues were collected adjacent to tumor tissues. All tissues were collected from the sample library for HCC and liver transplantation patients established by Shanghai General Hospital Affiliated to Shanghai Jiaotong University, School of Medicine. Eight cirrhosis tissues were obtained from diseased livers in liver transplantation. All of the five patients with HCC had a medical history of liver cirrhosis. All specimens were snap-frozen in liquid nitrogen and stored at −80 °C before using. In addition, another three pairs of HCC and adjacent normal tissues were collected for immunohistochemistry. The use of samples in scientific research has obtained the informed consent of patients. The research was permitted by Ethics Committee of Shanghai General Hospital.

### 2.2. RNA-Sequencing Analysis

Total RNA was extracted using the mirVana miRNA Isolation Kit (Ambion, Thermo Fisher Scientific, Waltham, MA, USA) following the manufacturer’s protocol. RNA integrity was evaluated using the Agilent 2100 Bioanalyzer (Agilent Technologies, Santa Clara, CA, USA). The samples with RNA Integrity Number (RIN) ≥ 7 were subjected to the subsequent analysis. The libraries were constructed using TruSeq Stranded Total RNA with Ribo-Zero Gold according to the manufacturer’s instructions. Then these libraries were sequenced on the Illumina sequencing platform (HiSeqTM 2500, Illumina, San Diego, CA, USA) and 150 bp/125 bp paired-end reads were generated. Sequenced reads were aligned to the human reference genome (hg38 assembly) using the TopHat2 [14]. The transcript abundance was obtained using bowtie2 and eXpress [15,16].

### 2.3. Public Database

In this study, a total of 158 FAM-related genes were obtained from Molecular Signatures Database (MSigDB) [17]. Gene expression data and clinical information of 371 HCC patients were downloaded from The Cancer Genome Atlas (TCGA) for construction of the prognostic signature, and GSE14520 dataset was obtained from Gene Expression Omnibus (GEO) for external validation [18]. Preliminary processing of data from TCGA and GEO was performed by “TCGAbiolinks Version 2.18.0” and “GEOquery Version 2.58.0” in R respectively. Expression data in two platforms of the GSE14520 was integrated by R package “sva Version3.38.0”. All of the three databases are publicly available.

### 2.4. Filtering of DEGs from Self-Building RNA-Sequencing Database

Differently expressed genes (DEGs) in the three comparisons (cirrhosis vs. paracancerous tissues, paracancerous tissues vs. cirrhotic HCC and cirrhosis vs. cirrhotic HCC) were filtered using Limma package Version 3.46.0 in R respectively. Expression data in RNA-sequencing were normalized by voom in Limma package. |Log2FC| > 1 and *p* < 0.05 were set as the cutoffs for the DEGs. Overall, DEGs in three comparisons were intersected using the venn diagram.

### 2.5. Mfuzz Cluster and Bioinformatic Analyses of DEGs from Self-Building RNA-Sequencing Database

The R package “Mfuzz Version 2.50.0” was used for cluster analysis of DEGs according to the order of HCC development, which gathered genes with similar expression trend into one group. Cirrhosis, para-carcinoma tissues and HCC were set as point1, point2 and point3, respectively. DEGs were divided into 8 clusters with a membership threshold of 0.25. GO enrichment and KEGG pathway analyses were performed to explore the potential biological processes, cellular components and molecular functions of the DEGs by the “clusterProfiler Version 3.18.1” package in R.

### 2.6. Construction of the FAM-Related Prognostic Signature Using Public MSigDB and TCGA Dataset

FAM-related DEGs were identified by overlapping DEGs and FAM gene sets from MSigDB. A total of 97 patients were selected from the TCGA-LIHC cohort for evaluating the prognostic value of FAM-related DEGs. Patient selection criteria were as follows: (1) have a follow-up time more than 30 days; (2) have a diagnosis of liver fibrosis. Subsequently, the prognostic signature was constructed by Lasso penalized Cox regression analysis using “glmnet Version4.1.4” package in R. A five-gene FAM-related prognostic signature with a corresponding coefficient was selected, and a risk score was calculated for each patient by formula as a follow-up (exp: value of FAM-related gene expression; coef: coefficient β for each gene given by LASSO algorithm):

$$Risk\;score=\overset{n}{\underset{i=1}{\sum }}\left(exp\left(i\right)*coef\left(i\right)\right)$$

Then, the 97 patients were divided into two groups based on the median of the risk score. To assess the performance of the five-gene prognostic signature, the Kaplan-Meier estimator curves were calculated using package “survival Version 3.3.1” in R. The time-dependent receiver operating characteristic (ROC) curve analysis was conducted by using the R package “ROCR Version 1.0.11”.

### 2.7. Independent Validation of Prognostic Gene Signature Using Multivariable Cox Regression Analysis and External Datasets

The risk score and other clinical variants, including age, sex, tumor grade, tumor stage, fibrosis status and AFP, were analyzed by univariable Cox regression analysis. A multivariable Cox regression analysis was applied to validate the value of the signature as an independent prognostic factor for cirrhotic HCC using the TCGA-LIHC cohort.

The GSE14520 dataset come from a GEO database was used to validate the performance of the FAM-related signature. A total of 221 patients in the GSE14520 datasets were selected for validation with the same criteria as 97 patients selected from the TCGA-LIHC cohort.

A composite nomogram was established based on all significant prognostic parameters determined through multivariate Cox regression analysis to predict the probability of 1-, 3-, 5-year OS using the “rms Version6.2.0” package in R. The calibration curve of a nomogram was utilized to vividly assess the consistency between its prediction probabilities and the actual observation. Time-dependent ROC curves of the nomogram were employed in compare with the American Joint Commission on Cancer (AJCC) stage.

### 2.8. Analyses of Tumor Immune Infiltration and Immune-Related Molecular Characteristics

To explore the relationship between FAM-related signature and tumor immune microenvironment, stromal, immune, and estimate scores were calculated for every patient selected from TCGA by an ESTIMATE algorithm with the “estimate Version 1.0.13” package by R software. A gene set consisting of 178 genes encoding immunomodulators and chemokines were obtained from TISIDB to verify the relationship between the prognostic signature and tumor immune microenvironment.

### 2.9. Immunohistochemistry

Clinical tissues were fixed in 4% paraformaldehyde for 12 h, dewaxed in xylene, rehydrated in descending series of alcohol (100, 95 and 75% alcohol), and heated in 0.01 M citrate buffer for 15–20 min. After cooling to room temperature, the tissues were washed with PBS, blocked with goat serum and left to stand at room temperature for 20 min. Next, the tissues were immunostained with primary antibodies against ADH1C (8081, Abclonal, Wuhan, China, 1:100) at room temperature for 1 h. The tissues were incubated with 30 μL secondary goat anti-rabbit immunoglobulin G (ab6721, 1:2000, Abcam, Cambridge, UK) for 1 h at room temperature. Thereafter, the tissues were treated with streptavidin-peroxidase, allowed to stand at 37 °C for 30 min, developed with 3,3′-diaminobenzidine tetrahydrochloride for 5–10 min, counterstained for 2 min, and differentiated with hydrochloric acid-ethanol. The tissues were observed under a microscope after conventional dehydration, clearing and mounting.

### 2.10. Cell Culture and Transfection

The hepatocellular carcinoma cell line HCCLM3 was obtained from Shanghai CellBank of Chinese Academy of Sciences (Shanghai, China) and cultured in Dulbecco’s modified Eagle’s medium (Gibco, Thermo Fisher Scientific, Waltham, MA, USA) containing 10% fetal bovine serum. Cells were incubated at 37 °C in a humidified atmosphere of 5% CO_2_.

HCCLM3 cell lines were seeded in a 6-well plate and cultured overnight before transfection. The siRNA targeting ADH1C was designed by Genomeditech (Shanghai, China). The siRNA was transfected with Lipofectamine 2000 (Invitrogen, Thermo Fisher Scientific, Waltham, MA, USA) according to the manufacturer’s protocol.

### 2.11. Quantitative Real-Time PCR (qRT-PCR)

Total RNAs from HCC cell lines were extracted using Trizol (Takara Biotechnology, Shiga, Japan). We used a reverse transcription kit (EnzyArtisan, Shanghai, China) to synthesize cDNA for subsequent PCR assay. QRT-PCR was performed with 2× S6 Universal SYBR qPCR Mix (EnzyArtisan, Shanghai, China). Relative mRNA expression levels were normalized to Actin and calculated by the 2^−ΔΔct^ method. The forward primer of ADH1C is ATGAGGCATGTGGCACAAGTGTC, and the reverse primer is GCGTCCAGTCAGTAGCAGCATAG.

### 2.12. CCK-8

Cells were cultured in 96-well plate with 3000 cells each well. The proliferation rate of HCCLM3 cells was detected by the Cell Counting Kit-8 assay (Dojindo Laboratories, Kumamoto, Japan). The absorbance at 450 nm of the experimental wells was measured at 24 h, 48 h and 72 h with an automatic microplate reader.

### 2.13. Ethynyldeoxyuridine (Edu) Analysis

Cells were cultured in 96-well plates and then incubated with the Cell-Light EdU Apollo 567 (RiboBio, Guangzhou, China) for 2 h. After treatment with 4% paraformaldehyde and 0.5% Triton X-100, DNA synthesis and cell proliferation were observed using a fluorescence microscope.

### 2.14. Wound-Healing Assay

HCCLM3 cells were cultured to 90% confluence in a 6-well plate after transfection. A scratch wound was generated using a sterile 200 μL pipette tip, and floating cells were removed by washing with 1× PBS. Scratched lines were photographed under 0 h, 24 h and 72 h.

### 2.15. Transwell Assay

This experiment was performed using 6.5 mm Transwell^®^ inserts of polycarbonate membranes with 8.0 µm pores (Corning, New York, NY, USA). 1 × 10^5^ cells were seeded in the upper chamber with 200 μL serum-free DMEM, while 600 μL DMEM containing 20% FBS was added to the bottom chamber. After 48 h, cells were fixed by paraformaldehyde and stained with 0.1% crystal violet and photographed.

### 2.16. Oil Red O Staining

For determination of lipid droplet, cells were cultured in a 6-well plate. Two days after transfection, cells were fixed in 4% PFA for 30 min and then incubated with Oil Red O for 15 min. The images were captured under optical microscope.

### 2.17. Statistical Analysis

Statistical analysis was performed in the R platform (v. 4.0.4). Lasso regression was performed for the establish of the prognostic model. The overall survival between subgroups was compared by the Kaplan–Meier method. Univariate and multivariate Cox proportional hazards regression analyses were used for assessing the prognosis value of the signature. Wilcoxon rank sum test was used to compare immune infiltration and immune-related gene expression between subgroups. Statistical significance was defined as a *p*-value of less than 0.05 (*p* < 0.05).

## 3. Results

### 3.1. Filtering of DEGs in Sequence of Cirrhosis, Paracancerous and HCC Tissues

**Figure 1:**
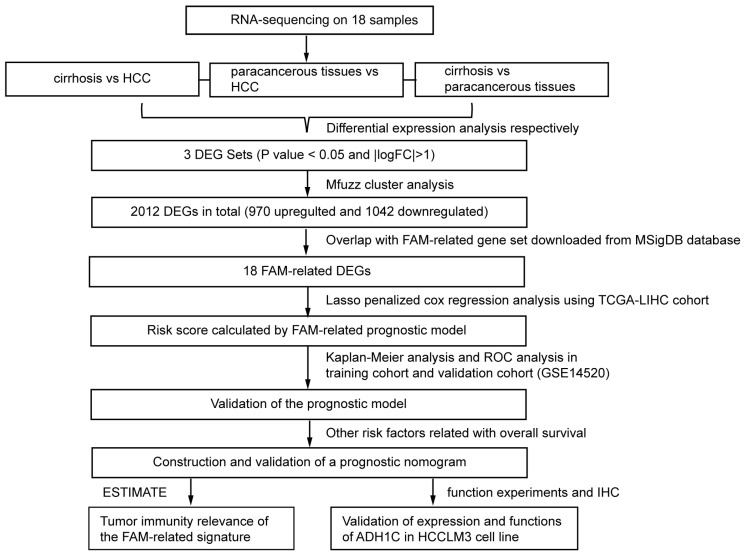
Flowchart presenting the process of establishing the gene signature and prognostic nomogram of cirrhotic HCC in this study.

**Figure 2:**
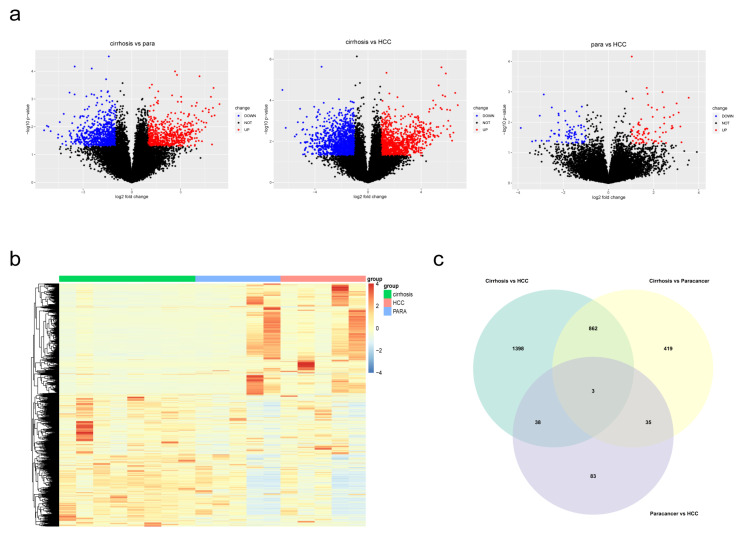
Identification of DEGs in the three comparisons. (**a**) Volcano plots revealed the DEGs between each comparison. (**b**) Heatmap of DEGs in cirrhosis, paracancerous tissues and HCC. (**c**) A Venn diagram revealed overall DEGs by combining three DEG sets.

The whole study flow is shown in Figure 1. Self-building RNA-sequencing database of cirrhosis, paracancerous and HCC tissues were analyzed. The DEGs of three comparisons were obtained respectively. As a consequence, 2301 DEGs (cirrhosis vs. HCC), 1319 DEGs (paracancerous tissues vs. cirrhosis) and 159 DEGs (paracancerous tissues vs. HCC) were identified. The volcano plots and heatmap of the 3 DEG sets were shown in Figure 2a,b. A total of 2838 overall DEGs were finally identified by combining the three DEG sets using a venn diagram (Figure 2c).

### 3.2. Mfuzz Cluster and Bioinformatic Analyses for Overall DEGs Screened from Self-Building RNA-Sequencing Database

**Figure 3:**
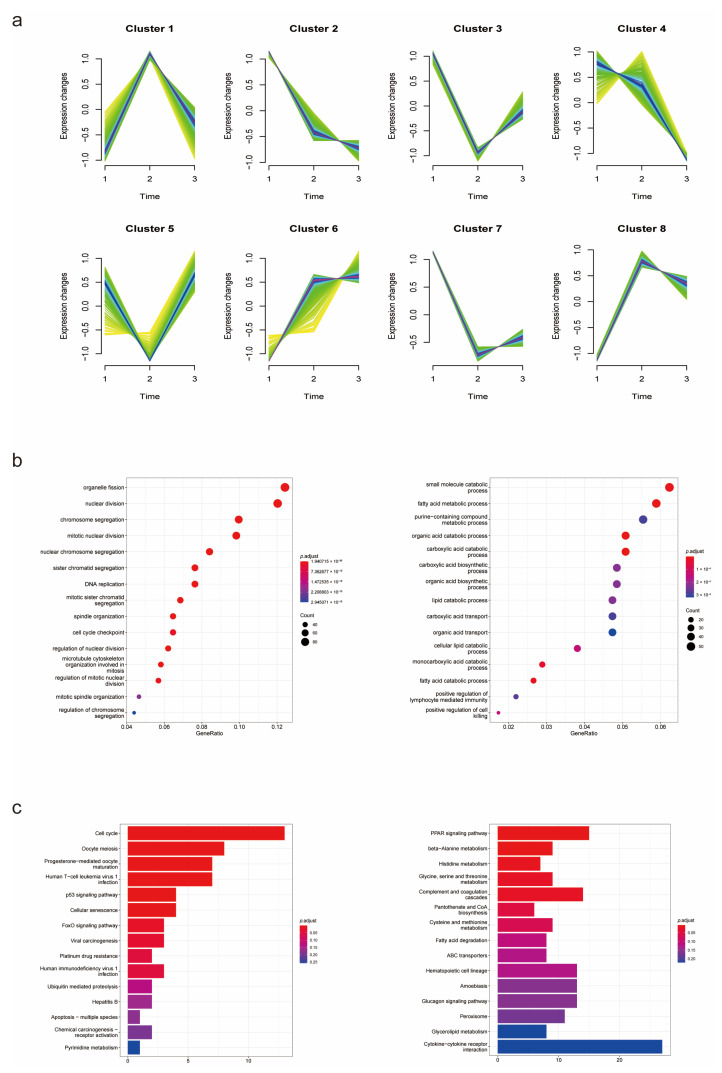
Mfuzz cluster and enrichment analyses of overall DEGs. (**a**) Mfuzz cluster analysis illustrated the changes in mRNA expression in the process of HCC development. (**b**,**c**) GO and KEGG enrichment analyses revealed potential functions of the DEGs and critical pathways in disease progression.

To identify genes which played crucial roles in the process from cirrhosis to HCC, the Mfuzz cluster analysis was performed to research the expression trends of DEGs in the three groups (Figure 3a). A total of 2838 overall DEGs obtained above were used as input for Mfuzz analysis. Seen as cirrhosis tissues with HCC background, paracancerous tissues of cirrhotic HCC patients could have been taken as an intermediate state between cirrhosis and HCC, so the order for Mfuzz analysis was as follows: 1. Cirrhosis, 2. Paracancerous tissues and 3. HCC. As a result, 8 clusters were obtained and then divided into 3 groups, including upregulated group (cluster 6,8), downregulated group (cluster 2,4,7) and discontinuous changing group (cluster 1,3,5). A total of 970 DEGs in upregulated groups and 1042 in downregulated groups were considered to be continuously changing in the development of HCC. To identify the biological functions of these DEGs and research the potential critical pathways in disease development, GO and KEGG enrichment analyses were performed by clusterProfiler (Figure 3b,c). DEGs increasing in this process were significantly enriched in cell cycle-related pathways, while downregulated DEGs were closely related to metabolism process. FAM ranked the top in these enriched pathways, indicating its critical function in the development of cirrhotic HCC.

### 3.3. Construction of the Five FAM-Related DEGs Prognosis Signature by Combining Self-Building RNA-Sequencing Database with Multidimensional Public Database

FAM was identified to participate in HCC development via enrichment analyses above. To better understand the role of FAM-related genes in cirrhotic HCC, a gene set composed of 158 mRNA involved in metabolism of fatty acids was downloaded from MSigDB dataset. A total of 18 FAM-related DEGs were identified by overlapping the DEGs obtained above and the FAM gene set. Names of FAM-related DEGs were listed in Table S1.

**Figure 4:**
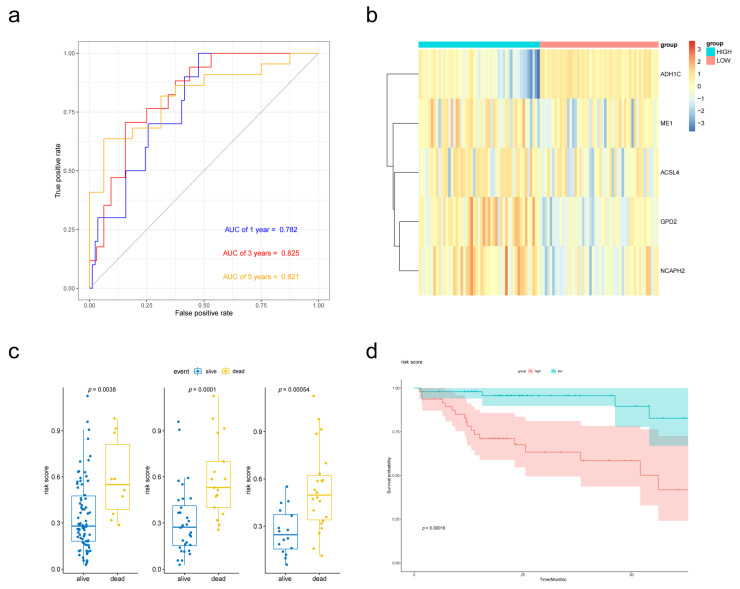
Validation of the prognostic performance of the FAM-related signature in the TCGA-LIHC dataset. (**a**) Time-dependent ROC curve of risk score model for 1-, 3- and 5-year overall survival predictions. (**b**) Heatmap of the five-gene expression in the TCGA cohort. (**c**) Boxplot of risk scores for different states. (**d**) Kaplan–Meier survival curves of the risk score model.

To establish the prognostic signature, a total of 97 samples with a follow-up period >30 days and a diagnosis of liver fibrosis were identified from TCGA. The clinical characteristics of selected patients were listed in Table 1. Then, Lasso penalized Cox regression analysis was performed with FAM-related DEGs, and a prognostic signature comprising 5 genes (ADH1C, ACSL4, GPD2, ME1, NCAPH2) was developed. The risk score was calculated as follows: Risk score = [(−0.08396789) × Expression value of ADH1C] + [(−0.01354980) × Expression value of ACSL4] + [(0.07405249) × Expression value of GPD2] + [(0.01765228) × Expression value of ME1] + [(0.08031334) × Expression value of NCAPH2]. Patients were divided into low- and high-risk groups according to the median risk score at 0.292565594. The gene expression was revealed in Figure 4b. The Kaplan–Meier survival curves revealed significantly poorer overall survival in the group with a higher risk score (*p* = 0.00019) (Figure 4d). A time-dependent ROC curve was performed to evaluate the prognostic values of the five-gene prognosis signature. The AUCs (area under the curves) for the 1-, 3-, 5-year OS predictions for the risk score were 0.782, 0.825 and 0.821, respectively (Figure 4a). Patients who survived at the 1st, 3rd and 5th years had significantly lower risk scores (Figure 4c).

### 3.4. Cox Proportional Hazards Regression Analysis of the Five FAM-Related DEGs Prognosis Signature

We further evaluated the effect of the FAM-related prognostic signature on the OS of cirrhotic HCC patients through univariate and multivariate cox regression. Clinical information of the 97 patients involved in this study was collected from TCGA. Univariate cox regression revealed that age, the AJCC stage and risk score were significantly correlated with HCC survival (*p* < 0.05). The multivariate Cox regression analysis uncovered that HCC patients with higher risk scores potentially have poorer prognosis (Table 2). Therefore, a high risk score was an independent risk indicator for cirrhotic HCC prognosis.

### 3.5. External Validation of the Prognostic Performance of the Five-Gene Signature Using GEO Database

**Figure 5:**
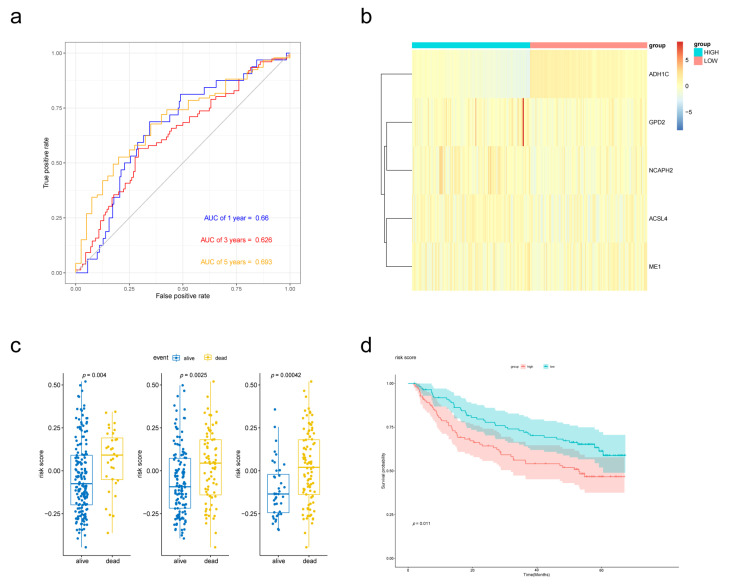
External validation of the prognostic performance of the five-gene signature in GSE14520 dataset. (**a**) Time-dependent ROC curve of risk score model for 1-, 3- and 5-year overall survival predictions. (**b**) Heat map of the five-gene expression in GSE14520 cohort. (**c**) Boxplot of risk scores for different states. (**d**) Kaplan–Meier survival curves of the risk score model.

To validate the performance of the prognostic signature with different data platforms, we downloaded GSE14520 which contained 223 cirrhotic HCC patients. Risk scores were calculated with the same formula and the patients were divided into two groups based on the median risk score. Patients with lower risk scores had obviously better OS according to the Kaplan–Meier survival curves (Figure 5d). Then, prognostic power was evaluated by time-dependent ROC and the Wilcoxon Rank Sum Test. Risk scores were significantly different between the alive group and dead group (Figure 5c). *p* value of 1-, 3- and 5-year overall survival predictions for the signature were 0.004, 0.0025 and 0.00042 respectively, which indicated good performance of the FAM-related signature at predicting prognosis in cirrhotic HCC patients.

### 3.6. Building and Validating a Novel Predictive Nomogram including of the Five FAM-Related DEGs Prognosis Signature

**Figure 6:**
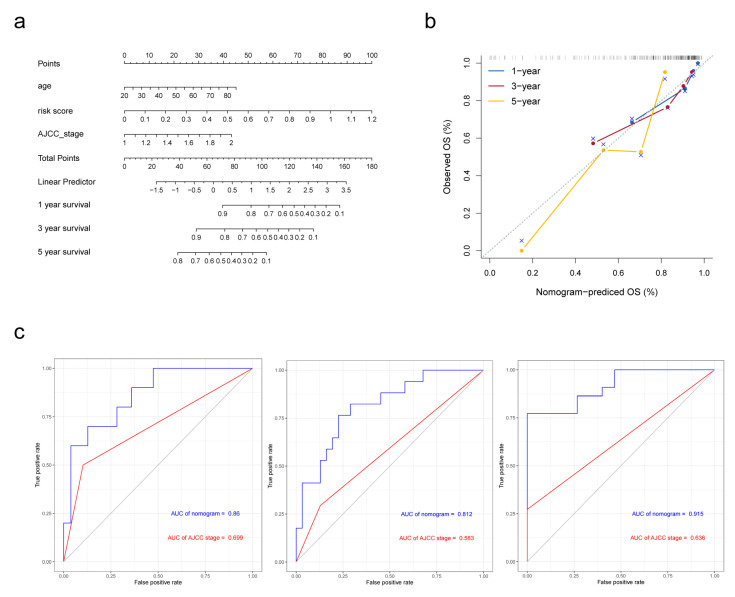
Establishment and validation of the nomogram in predicting overall survival of cirrhotic HCC in the TCGA-LIHC. (**a**) A prognostic nomogram predicting 1-, 3-, and 5-year overall survival rates of HCC. (**b**) Calibration plot of the nomogram for predicting the probability of survival at 1, 3, and 5 years. (**c**) Time-dependent ROC curve of the nomogram for 1-, 3-, and 5-year overall survival predictions comparing with AJCC stage.

In Cox proportional hazards regression analysis above, risk score, AJCC stage and age were filtered to be associated with OS. For better prediction of cirrhotic HCC patients’ prognosis, a novel nomogram combing these two clinical parameters and FAM-related signature was established (Figure 6a) using data of 93 cirrhotic HCC patients from TCGA-LIHC cohort, as 4 in 97 patients were excluded for the lack of clinical information. Calibration plots were performed for the validation of the nomogram (Figure 6b). The AUCs of the 1-, 3-, and 5-year OS predictions for the nomogram were 0.86, 0.812 and 0.915 (Figure 6c), respectively, which were significantly higher than the AUCs for AJCC stage, indicating that the nomogram performed better in predicting the prognosis of cirrhotic HCC.

### 3.7. Tumor Immunity Relevance of the FAM-Related Signature and Expression of Immune-Related Genes

**Figure 7:**
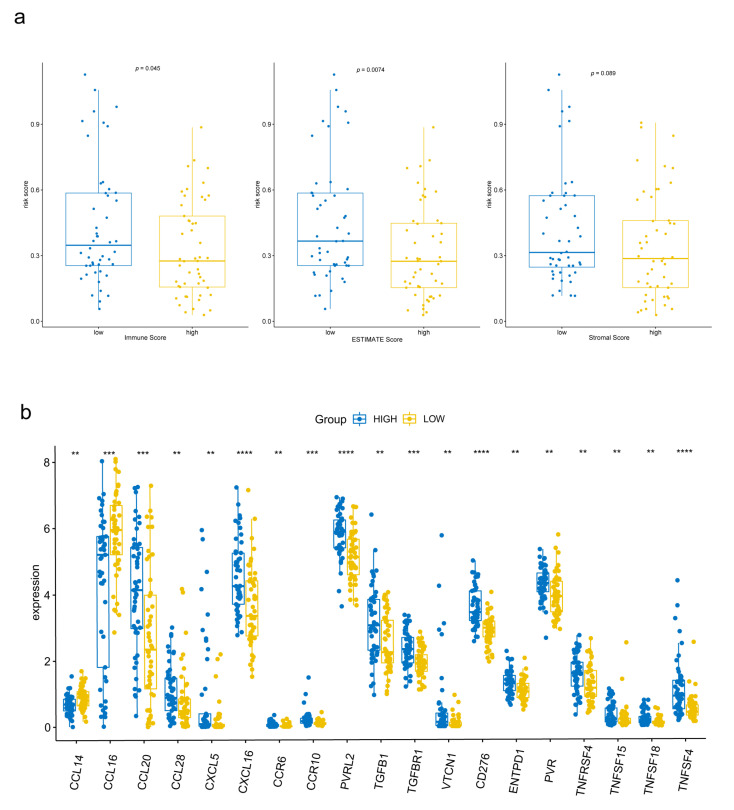
Tumor immunity relevance of the FAM-related signature. (**a**) Relationship among immune scores, ESTIMATE scores, stromal scores and risk scores. (**b**) Differently expressed immune-related genes between the high-risk group and low-risk group. ** *p* < 0.01, *** *p* < 0.001, **** *p* < 0.0001.

To explore the relationship between the signature and tumor immune microenvironment, we performed ESTIMATE to calculate immune scores, ESTIMATE scores, and stromal scores for 97 cirrhotic HCC patients from TCGA. Patients were divided into high group and low group according to the scores, respectively. As a result, patients with lower immune scores and ESTIMATE scores had higher risk scores (Figure 7a). Also, the expression of genes encoding immunomodulators and chemokines were compared between high-risk and low-risk group. In 178 genes we downloaded from TISIDB database, 127 could be found in TCGA expression matrix and 30 were differently expressed between two groups (*p* < 0.05). The most significant ones (*p* < 0.01) were shown in Figure 7b.

### 3.8. Construction of FAM-Related lncRNA-mRNA Network

**Figure 8:**
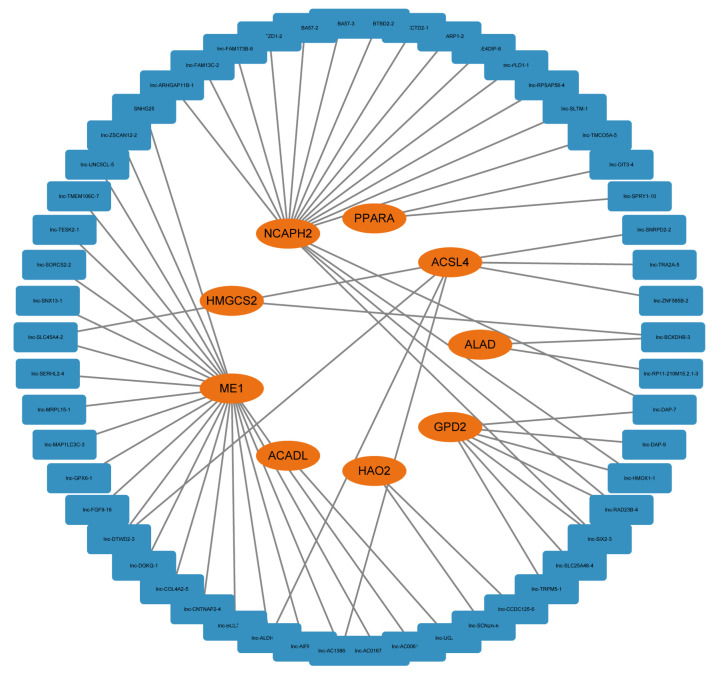
A FAM-related lncRNA-mRNA regulatory network consisting of 9 mRNAs and 54 lncRNAs.

Long non-coding RNA (lncRNA) plays an important role in gene regulation and cancer development. To explore lncRNA, which is related with FAM and participates in HCC progression, we extracted the sequence results of lncRNA and performed difference analysis and Mfuzz cluster analysis on it (Figure S2a,b). A total of 2107 dynamic changing lncRNAs were obtained, including 960 upregulated ones and 1147 downregulated ones (Figure S2c and Table S3). Pearson correlation coefficients were calculated between FAM-related genes and lncRNAs in order to explore the potential regulatory relationship. Correlation coefficients >0.9 and *p* < 0.001 were set as threshold to identify FAM-related lncRNA. Finally, a FAM-related regulatory network containing 9 mRNAs and 54 lncRNAs were established (Figure 8). Correlation coefficient between lncRNA and mRNA was established in Table S2.

### 3.9. Validation of Expression and Function of ADH1C by IHC and Cell Experiments

**Figure 9:**
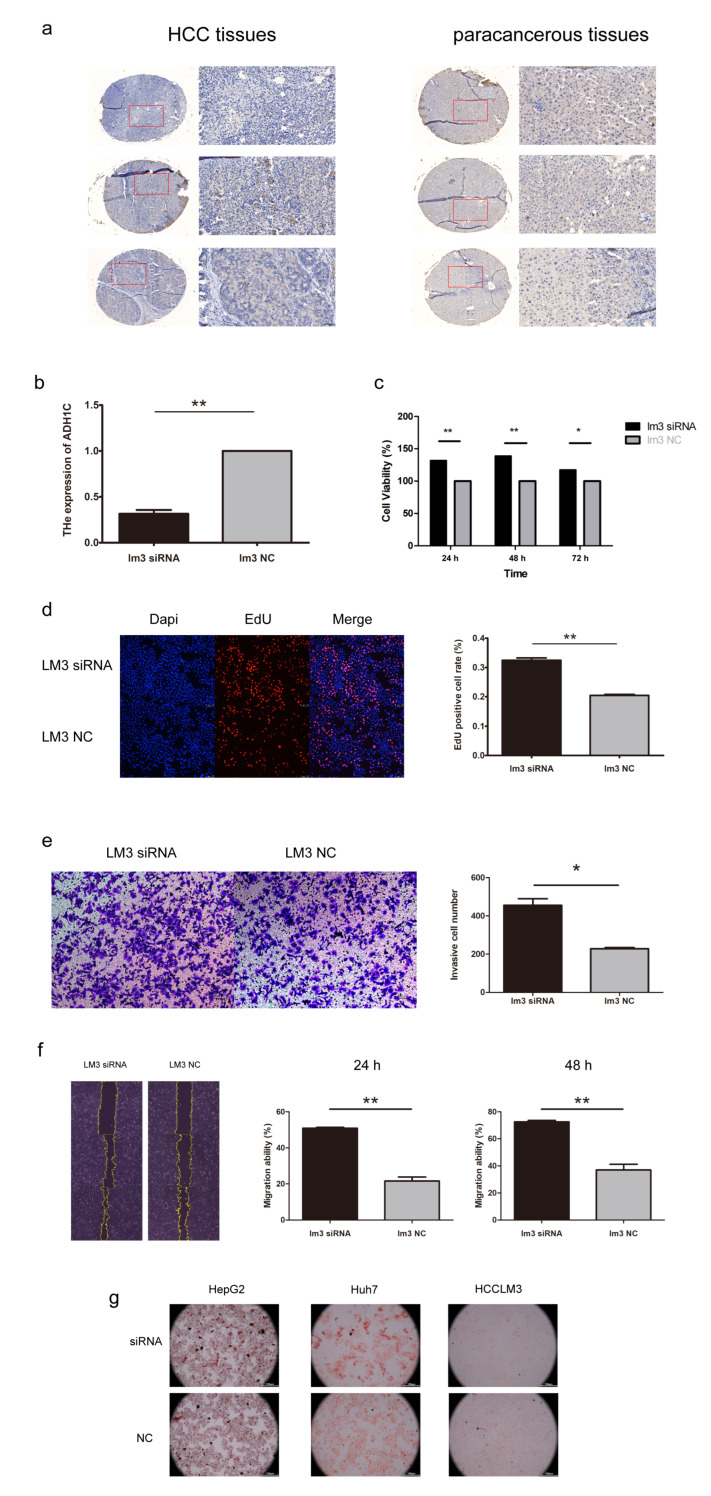
The expression and function of ADH1C in HCC. (**a**) Immunohistochemistry (IHC) staining of ADH1C in HCC and adjacent normal tissues. (**b**) The efficiency of siRNA targeting ADH1C. (**c**,**d**) CCK-8 and EdU assay indicated that ADH1C knockdown promoted proliferation of HCCLM3. (**e**,**f**) Transwell and the wound healing assay indicated that ADH1C knockdown promoted migration of HCCLM3. (**g**) Oil red O staining of HCC cell lines to check lipid droplets’ accumulation. * *p* < 0.05, ** *p* < 0.01.

We performed survival analysis on the 3 genes selected in the signature. ADH1C was identified as a protective factor and may play a critical role in HCC progression (Figure S1). Then, the expression of ADH1C was verified in 3 pairs of HCC and adjacent normal tissues using IHC. As a result, ADH1C expression was significantly downregulated in HCC tissues on protein level (Figure 9a). To further explore the function of ADH1C in HCC development, siRNA was transfected into the HCCLM3 cell line to knock down ADH1C (Figure 9b). The CCK8 and EdU assays illustrated that a knockdown of ADH1C can significantly enhance the proliferation of HCCLM3 transfected with siRNA (Figure 9c,d). In addition, we observed that ADH1C silencing effectively enhanced the migration ability of HCCLM3 cells by performing transwell and wound healing assays (Figure 9e,f). In brief, knockdown of ADH1C effectively promoted the proliferation and migration of HCCLM3 cells. In addition, Oil red O staining was performed in three HCC cell lines to check the lipid droplets’ accumulation after transfection (Figure 9g).

## 4. Discussion

Liver cancer is one of the most common and the most mortal cancers worldwide, which causes more than 830,000 deaths in 2020 [1]. HCC occupies the majority of liver cancer. Cirrhosis is an important precancerous lesion for HCC, with 90% patients developed HCC under a background of cirrhosis [19]. Exploring the detailed mechanism of cirrhosis to HCC would be useful to clarify carcinogenesis of HCC and may benefit to HCC therapy.

In this study, to further explain the molecular mechanism of cirrhosis to HCC, we performed RNA-sequencing on cirrhosis, paracancerous and HCC tissues. Combing with Mfuzz clustering analysis, a self-building RNA-sequencing database of HCC development was established. GO and KEGG enrichment analyses were employed using 2012 continuously change DEGs in this database for function analysis. Metabolism-associated terms, especially FAM-related ones, were significantly enriched in downregulated DEGs, indicating their important position in promoting cirrhosis to HCC. FAM-related DEGs were identified by overlapping DEGs with a hallmark gene set from MSigDB database. A total of 97 HCC patients with cirrhosis were selected from TCGA, and 5 genes (ADH1C, ACSL4, GPD2, ME1, NCAPH2) were selected to construct a FAM-related prognosis signature via Lasso–Cox regression using this cohort. The prognostic efficiency of the FAM-related signature was verified via Kaplan–Meier survival curve analysis and ROC analysis in both internal and external datasets. Multivariate Cox regression analysis indicated that the FAM-related signature was an independent prognostic factor of cirrhotic HCC. A nomogram consisting of the gene signature and clinical parameters was established, which performed better in predicting OS of cirrhotic HCC patients. ESTIMATE algorithm analysis was performed and the expression of immune-related genes was compared, which indicated the relevance between a FAM-related signature and immune. Finally, ADH1C was selected and function experiments were performed using the HCC cell line.

Differently expressed genes between three comparisons were identified respectively and then combined in this study. We observed that the number of DEGs in the comparison of paracancerous tissues and HCC was respectively small. Similar analyses were performed on data from the TCGA dataset (Figure S3), and due to the fact that the number of DEGs in TCGA was significantly higher than that found in our data, the following were possible causes: (1) HCC and paracancerous tissues were taken from the same group of HCC patients who shared a history of cirrhosis. Comparatively, the HCC and paracancerous tissues in TCGA are not identical. This may also be the cause of the relatively significant discrepancy in the cirrhosis group, which was compiled from the remaining 8 patients. (2) Paracancerous tissues were gathered close to the site of the tumor, which could be influenced by satellite lesions, taking into account the impaired liver function of cirrhotic HCC patients.

Abnormality in FAM was proved to participate in the progression of multiple cancers, including lung cancer, ovarian cancer and HCC [20,21,22]. Cancer cells can redirect metabolic pathways to meet energy demands through the regulation of FAM [23]. A variety of FAM-related genes were confirmed to influence the proliferation and metastasis of the HCC cell. For example, CD147 reprogrammed FAM in HCC cells through Akt/mTOR/SREBP1c and P38/PPARα pathways [20], while ACADM was reported to influence HCC via aberrant CAV1/SREBP1 signaling [24]. Several FAM-related gene prognostic signatures have been established, which contributed to the understanding and therapies of HCC [25,26]. FAM was also known to participate in chronic liver diseases and may influence cirrhosis progression [27]. For instance, CPT1A, a FAM-related gene, was reported to be critical in the activation of hepatic stellate cells and was implicated in the development of liver cirrhosis via fatty acid oxidation [28]. It was also reported that the level of fatty acid in erythrocyte membrane had a relationship with HCC risk in cirrhotic patients, indicating the function of FAM in HCC development [29]. However, the specific role of FAM in cirrhosis to HCC process, especially in transcriptome level, remained unclear. In our research, we identified FAM to play a key role in cirrhotic HCC development via RNA-sequencing and multiple bioinformatics analyses, which contributed to the understanding of the molecular mechanism among FAM, cirrhosis and HCC.

The significant role of metabolism reprogramming, including FAM, in tumor-immune microenvironment has been confirmed in previous studies [30,31]. Abnormal metabolism features of tumor cells cause hypoxia and nutrient depletion in tumor microenvironment, thus influence the composition and function of the infiltrated immune cells [32,33]. Our results supported this conclusion, as the risk scores calculated by a FAM-related signature were significantly related with immune infiltrate. We also revealed that several immune-related genes were differently expressed in two subgroups, indicating genes in the signature may act as targets for immune therapy of HCC in the future.

Long non-coding RNA (lncRNA) participates in the biological process through multiple ways, including regulating protein-coding RNA. Abnormal changes in lncRNA may influence the development of different kinds of cancers, including HCC [34]. Recent studies verified the critical role of lncRNA in HCC biology [35,36,37]. To explore lncRNA related with FAM and its influences in HCC development, we identified continuous changing lncRNAs from cirrhosis to HCC, and a FAM-related lncRNA-mRNA network was established by correlation analysis. LncRNA in this network had a potential ability in regulating FAM process and HCC development.

3 of the 5 genes in the signature were reported to be associated with HCC. ACSL4 (acyl-CoA synthetase long chain family member 4) is a ferroptosis-related gene and is closely involved in the progression of multiple cancers, such as breast cancer, ovarian cancer and glioma [38,39,40]. In HCC, ACSL4 was reported to promote cell growth and metastasis via the c-Myc/SREBP1 pathway and ERK/FBW7/c-Myc axis [41,42]. ACSL4 was also identified as a predictive biomarker of sorafenib sensitivity in HCC [43]. ME1 (malic enzyme 1) encodes a NADP-dependent enzyme that generates NADPH for fatty acid biosynthesis. In gastric cancer, ME1 promotes cell growth and metastasis by regulating NADPH homeostasis [44]. In addition, changes in phosphorylation and acetylation of ME1 can also affect colorectal tumorigenesis [45]. ME1 was detected as a biomark for HCC by several bioinformatic studies [46,47], and its function in proliferation, migration, and invasion has been confirmed in the HCC cell line [48]. GPD2 (glycerol-3-phosphate dehydrogenase 2) participates in the glycolysis process and ferroptosis defense, and is reported to affect the mitochondrial energy metabolism and the stemness in the HuH7 cell line [49,50]. NCAPH2 encodes one of the non-SMC subunits of the condensin II complex, which plays an essential role in mitotic chromosome assembly. It was identified as a novel driver gene in cervical cancer by a comprehensive analysis research [51]. The role of GPD2 and NCAPH2 in HCC progress is still unclear and needs more research.

Alcohol dehydrogenase (ADH) is a metabolizing enzyme family, which participates in alcohol metabolism by transforming ethanol to acetaldehyde. Change of enzymes in this family is reported to have an effect on HCC prognosis [52]. As a member of the ADH family, ADH1C is involved in the development of several cancers. In colorectal cancer, ADH1C is reported to inhibit progression through the serine metabolic pathway [53], and is a protective factor for CRC patients via bioinformatics analysis [54]. Previous studies have uncovered that ADH1C is downregulated in HCC, and overexpression of ADH1C indicates better prognosis for HCC patients according to bioinformatics analyses [55,56]. In addition, ADH1C was identified in a study to be a key gene in tumor microenvironments in HCC [57], and in consideration of its role as an important regulator of intestinal inflammation [58], the effect of ADH1C in immunity deserves further consideration. Most researches about ADH1C in HCC stopped at bioinformatics analyses. In our study, we verified the expression of ADH1C in HCC via IHC, revealing that ADH1C was differently expressed both in mRNA and protein level. Afterwards, we confirmed the ability of ADH1C in inhibiting cell migration and proliferation by silencing it in the HCC cell line. In addition, Oil red O staining was performed in Huh7, HepG2 and HCCLM3 to check the function of ADH1C in the fatty acid metabolism. No discernible change was found in our assay, which indicated that though ADH1C was involved in the Fatty Acid Degradation (hsa00071) Pathway and classified as a FAM-related gene, it may only have a small direct impact on the fatty acid content of HCC cell lines. Our experiments laid the foundation for further study on ADH1C in HCC.

In this study, we filtered DEGs in developing the process of cirrhotic HCC by combining the self-building RNA-sequencing database with public databases. However, there are still some limitations in our study. Cell experiments were only performed on ADH1C, and the functions of other genes in the signature still need to be verified. In addition, the detailed mechanism of ADH1C in HCC development remains unclear, which will be explored in our future work.

## 5. Conclusions

In summary, we described the genetic changes in the transition from cirrhosis to cirrhotic HCC by RNA-sequencing and identified the fatty acid metabolism as a key pathway in this process. A FAM-related gene signature was established for prognosis of cirrhotic HCC, and the function of ADH1C, a gene in this signature, was verified via cell experiments. Our study revealed the molecular relationship among FAM, cirrhosis and HCC at the transcriptome level.

## Figures and Tables

**Table 1:** Clinical features of selected TCGA-LIHC patients.

Age		58.4 ± 11.5
Follow-up time(month)		31.8 ± 22.9
Survival status		
	Alive	75 (77.3%)
	Dead	22 (22.6%)
Gender		
	Male	77 (79.4%)
	Female	20 (20.6%)
Grade		
	G1	11 (11.3%)
	G2	48 (49.5%)
	G3	34 (35.1%)
	G4	3 (3.1%)
	unknown	1 (1.0%)
AJCC stage		
	Stage I	55 (56.7%)
	Stage II	25 (25.8%)
	Stage III	13 (13.4%)
	unknown	4 (4.1%)
T classification		
	T1	57 (58.8%)
	T2	27 (27.8%)
	T3	11 (11.3%)
	T4	1 (1.0%)
	unknown	1 (1.0%)
N classification		
	N0	73 (75.3%)
	N1	1 (1.0%)
	NX	22 (22.7%)
	unknown	1 (1.0%)
M classification		
	M0	77 (79.4%)
	MX	20 (20.6%)
Fibrosis status		
	Fibrous Speta	24 (24.7%)
	Incomplete Cirrhosis	8 (8.2%)
	Established Cirrhosis	65 (67.0%)
Vascular invasion		
	No	64 (66.0%)
	Macro	3 (3.1%)
	Micro	25 (25.8%)
	unknown	5 (5.2%)
Cancer status		
	With tumor	42 (43.3%)
	Tumor free	35 (36.1%)
	unknown	20 (20.1%)
History of radiation treatment		
	Yes	4 (4.1%)
	No	76 (78.4%)
	unknown	17 (17.5%)

**Table 2:** Univariate and multivariate Cox regression analysis of TCGA-LIHC patients.

Characteristics	Univariate Cox		Multivariate Cox	
	*p*	HR (95%CI)	*p*	HR (95%CI)
Risk score	0.00011	18 (4.1–75)	0.000033	62 (8.8–440)
Age (<65/≥65)	0.028	0.39 (0.17–0.9)	0.084	0.43 (0.17–1.1)
Sex (female/male)	0.45	1.6 (0.47–5.4)	0.37	1.8 (0.48–7.1)
Grade (G1+G2/G3+G4)	0.54	0.77 (0.33–1.8)	0.059	0.36 (0.13–1)
AJCC stage (I+II/III+IV)	0.0064	0.26 (0.097–0.68)	0.0063	0.17 (0.046–0.6)
Fibrosis level	0.85	0.92 (0.37–2.3)	0.038	0.29 (0.091–0.94)
AFP (<400/≥400)	0.93	0.95 (0.32–2.8)	0.37	1.8 (0.51–6.1)

## Data Availability

The raw sequence data reported in this paper have been deposited in the Genome Sequence Archive (Genomics, Proteomics & Bioinformatics 2021) in National Genomics Data Center (Nucleic Acids Res 2022), China National Center for Bioinformation/Beijing Institute of Genomics, Chinese Academy of Sciences (GSA-Human: HRA003448) that are publicly accessible at https://ngdc.cncb.ac.cn/gsa-human (accessed on 29 October 2021).

## Supplementary Materials

The following supporting information can be downloaded at: www.mdpi.com/article/10.3390/biom13010141/s1, Figure S1: Kaplan-Meier survival curves of the 5 genes in FAM-related prognostic signature; Figure S2: Identification of dynamic changing lncRNAs in process of cirrhosis to HCC; Figure S3: Volcano plots revealed the differently expressed mRNAs between paracancerous tissues and HCC tissues in TCGA-LIHC cohort; Table S1: List of FAM-related DEGs. Table S2: Correlation between FAM-related genes and lncRNAs; Table S3: Differently expressed lncRNA.
