# Supplementary material for: HCC: RNA-Sequencing in Cirrhosis
Source: Biomolecules. 2023 Jan 10;13(1):141. doi: 10.3390/biom13010141 (PMC9855755; doi:10.3390/biom13010141)
Supplement: Supplementary file 1 [file biomolecules-13-00141-s001.zip › biomolecules-2031146 supp for final-2.pdf]

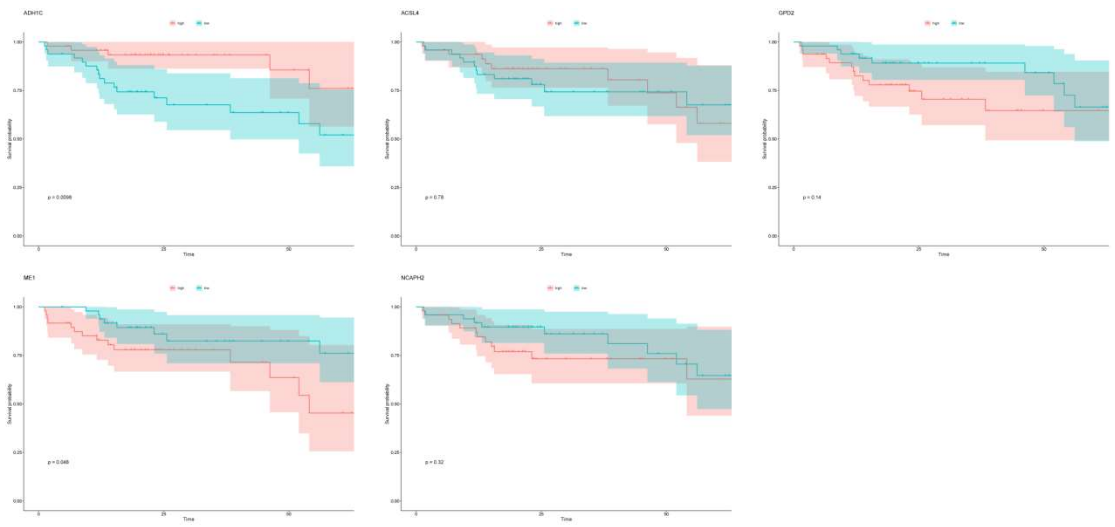

Figure S1: Kaplan-Meier survival curves of the 5 genes in FAM-related prognostic signature

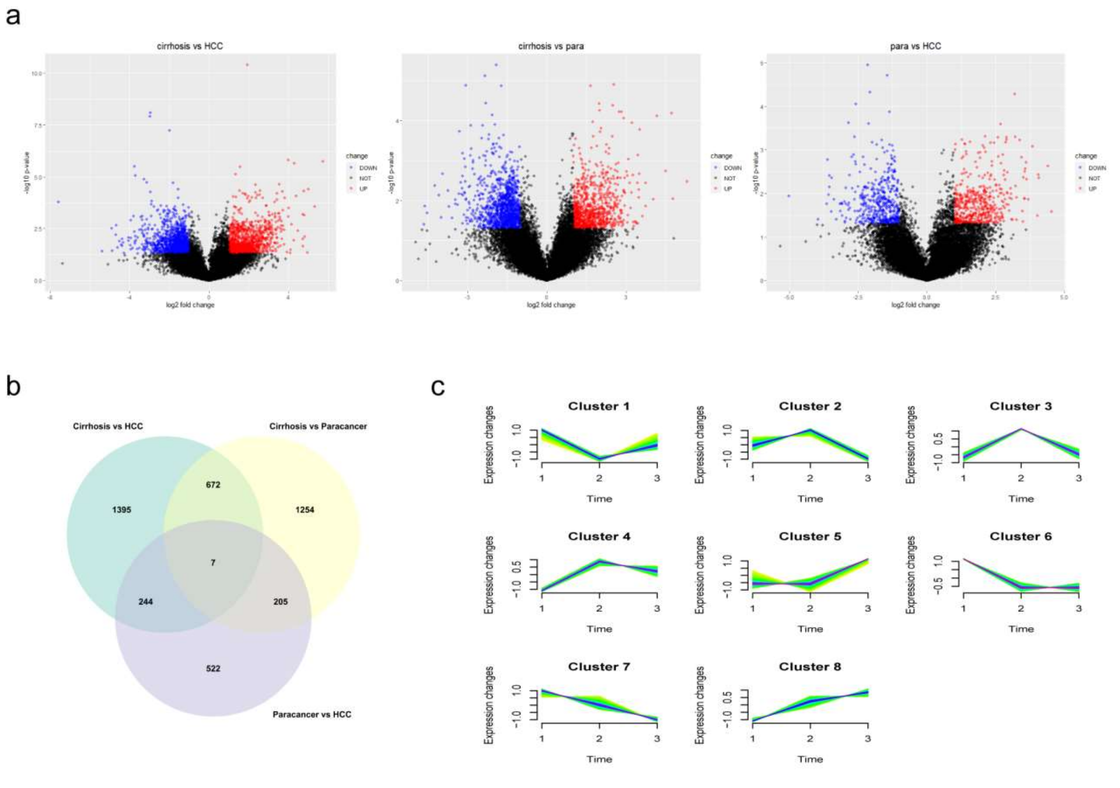

Figure S2: Identification of dynamic changing lncRNAs in process of cirrhosis to HCC. **a** Volcano plots revealed the differently expressed lncRNAs between each comparison. **b** Venn diagram revealed overall differently expressed lncRNAs by combining three DEG sets. **c** Mfuzz cluster analysis illustrated the changes in lncRNA expression in the process of HCC development.

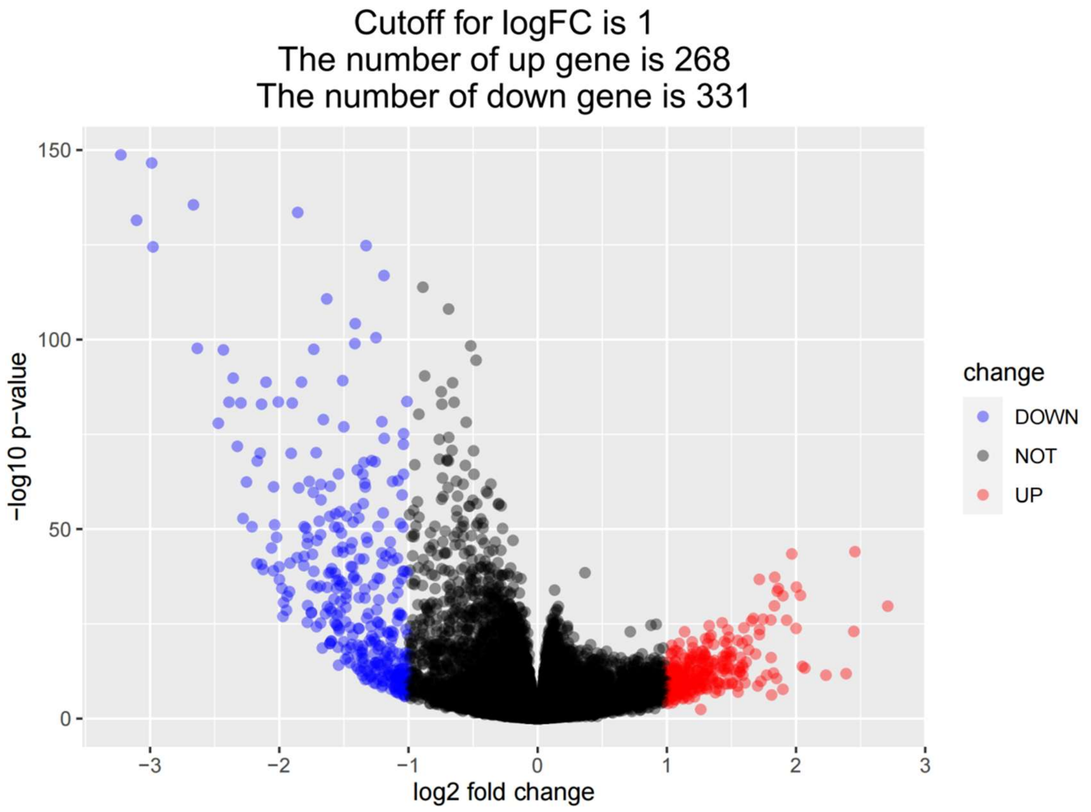

Figure S3: Volcano plots revealed the differently expressed mRNAs between paracancerous tissues and HCC tissues in TCGA-LIHC cohort.

Table S1: List of FAM-related DEGs

Symbol
GPD2
ME1
NCAPH2
ACSL4
ALAD
ADH1C
HAO2
ACADL
RDH16
GPD1
HMGCS2
TDO2
ACOX1
MLYCD
GABARAPL1
BCKDHB
ACSL5

PPARA

Table S2: Correlation between FAM-related genes and lncRNAs

mRNA	lncRNA	correlation coefficient	p_value
ALAD	lnc-BCKDHB-3	0.912494143	1.30988E-07
	lnc-RP11-210M15.2.1-3	0.920863211	6.021E-08
	lnc-DAP-7	0.951585195	1.30344E-09
	lnc-DAP-9	0.920522339	6.2248E-08
	lnc-HMOX1-1	0.913759357	1.17058E-07
GPD2	lnc-RAD23B-4	0.938714781	8.24748E-09
	lnc-SIX2-3	0.928456681	2.75283E-08
	lnc-SLC25A48-4	0.900292195	3.57787E-07
	lnc-TRPM5-1	0.918575589	7.50727E-08
HAO2	lnc-CCDC125-6	0.921059924	5.90604E-08
	lnc-SCN2A-6	0.906332234	2.21314E-07
ACADL	lnc-UGDH-3	0.919541069	6.84524E-08
	lnc-AC006156.1-12	0.963244891	1.49225E-10
	lnc-AC016757.3.1-1	0.968177062	4.78608E-11
	lnc-AC138647.1.1-5	0.928851295	2.63702E-08
	lnc-AIFM3-1	0.905718611	2.3272E-07
	lnc-ALDH8A1-3	0.977856458	2.71181E-12
	lnc-BCL2L10-2	0.92231745	5.21506E-08
	lnc-CNTNAP2-4	0.906428655	2.19567E-07
	lnc-COL4A2-5	0.920037342	6.52507E-08
	lnc-DGKQ-1	0.952671149	1.09086E-09
	lnc-DTWD2-3	0.945979779	3.07595E-09
	lnc-FGF9-16	0.926395017	3.43253E-08
ME1	lnc-GPX6-1	0.95964237	3.11715E-10
	lnc-MAP1LC3C-3	0.962918839	1.59985E-10
	lnc-MRPL15-1	0.906876403	2.11607E-07
	lnc-SERHL2-4	0.9194497	6.90564E-08
	lnc-SLC45A4-2	0.9496661	1.76808E-09
	lnc-SNX13-1	0.956124423	6.01588E-10
	lnc-SORCS2-2	0.925270848	3.8611E-08
	lnc-TESK2-1	0.913948944	1.15086E-07
	lnc-TMEM106C-7	0.967194055	6.08601E-11
	lnc-UNC5CL-5	0.903747984	2.72856E-07
	lnc-ZSCAN12-2	0.94033027	6.69493E-09
	SNHG25	0.929229652	2.52996E-08
HMGCS2	lnc-BCKDHB-3	0.931578791	1.94579E-08
	lnc-ARHGAP11B-1	0.951145287	1.3993E-09
NCAPH2	lnc-DAP-7	0.931414194	1.98251E-08
	lnc-FAM13C-2	0.946000444	3.06675E-09
	lnc-FAM173B-6	0.906677874	2.15105E-07

	lnc-FZD1-2	0.922756637	4.99084E-08
	lnc-HMOX1-1	0.945729575	3.18925E-09
	lnc-IBA57-2	0.922666102	5.03636E-08
	lnc-IBA57-3	0.944557078	3.76991E-09
	lnc-KBTBD2-2	0.903745065	2.7292E-07
	lnc-KCTD2-1	0.914672428	1.0782E-07
	lnc-LARP1-2	0.931490148	1.96549E-08
	lnc-PDE4DIP-6	0.90217115	3.09152E-07
	lnc-PLD1-1	0.902058825	3.1189E-07
	lnc-RAD23B-4	0.951497982	1.32197E-09
	lnc-RPSAP58-4	0.921373268	5.72684E-08
	lnc-SIX2-3	0.967312962	5.91397E-11
	lnc-SLTM-1	0.907939772	1.93697E-07
	lnc-TMCO5A-5	0.909754794	1.66145E-07
PPARA	lnc-OIT3-4	0.907896074	1.94406E-07
	lnc-SPRY1-10	0.907775651	1.96372E-07
	lnc-AC138647.1.1-5	0.936595712	1.07519E-08
	lnc-ALDH8A1-3	0.909648463	1.6766E-07
	lnc-DTWD2-3	0.900901995	3.41326E-07
ACSL4	lnc-SLC45A4-2	0.904138704	2.64455E-07
	lnc-SNRPD2-2	0.909200517	1.74174E-07
	lnc-TRA2A-5	0.940085899	6.91206E-09
	lnc-ZNF585B-2	0.920523073	6.22436E-08
